# Treatment Strategy for Esophageal Squamous Cell Carcinoma With Endoscopic Intramural Metastasis

**DOI:** 10.7759/cureus.23028

**Published:** 2022-03-10

**Authors:** Akihiko Okamura, Shoichi Yoshimizu, Jun Kanamori, Yu Imamura, Takao Asari, Izuma Nakayama, Mariko Ogura, Akiyoshi Ishiyama, Toshiyuki Yoshio, Keisho Chin, Junko Fujisaki, Masayuki Watanabe

**Affiliations:** 1 Department of Gastroenterological Surgery, Cancer Institute Hospital of Japanese Foundation for Cancer Research, Tokyo, JPN; 2 Department of Gastroenterology, Cancer Institute Hospital of Japanese Foundation for Cancer Research, Tokyo, JPN; 3 Department of Radiation Oncology, Cancer Institute Hospital of Japanese Foundation for Cancer Research, Tokyo, JPN

**Keywords:** esophageal cancer, neoadjuvant chemotherapy, endoscopic diagnosis, intramural metastasis, esophageal squamous cell carcinoma

## Abstract

Purpose: Intramural metastasis (IM) in esophageal squamous cell carcinoma (ESCC) is sometimes found, and the prognosis of ESCC patients with pathologically diagnosed IM is known to be dismal. However, there are few reports on ESCC patients with clinically diagnosed IM.

Methods: This study assessed 2,772 ESCC patients who underwent endoscopy for initial evaluation. Among them, 85 patients (3.1%) were diagnosed with endoscopic IM. In this study, we investigated these patients’ characteristics, survival among the groups stratified by the treatment modalities, and survival predictors.

Results: Of 85 patients, 76 (89.4%) had T3 or T4 tumors, 73 (85.9%) had nodal metastases, and 36 (42.4%) had M1 diseases. Curative-intent treatment could be given to 63 patients (74.1%) with a median survival time (MST) of 15.6 months (95% CI: 10.7-20.4). As initial treatment, upfront surgery (US), neoadjuvant chemotherapy (NAC) using cisplatin and 5-fluorouracil (CF), neoadjuvant chemoradiotherapy, and definitive chemoradiotherapy (dCRT) were given to 17 (27.0%), 27 (42.9%), 2 (3.2%), and 17 patients (27.0%), respectively. dCRT was preferred for T4 tumors compared with US or NAC (P = 0.02). The MST of US and NAC patients was 19.3 (95% CI: 12.9-25.6) and 23.4 months (95% CI: 9.4-37.4), respectively. No significant difference was noted between US and NAC patients (P= 0.89).

Conclusion: The prognosis of ESCC patients with endoscopic IM is poor even if curative-intent treatment is done. Moreover, no significant survival benefit of NAC with CF for these patients was observed when compared with US.

## Introduction

Pathologically diagnosed intramural metastasis (IM) of esophageal squamous cell carcinoma (ESCC) is sometimes found with an incidence rate of 5.5-16.6%, and the prognosis of these patients was suggested to be dismal [1-7]. In our previous study, pathological IM has shown to be an indicator of lymphatic invasion and advanced cancer in ESCC patients [7]. Previous reports on IM were mainly on its pathological diagnosis after surgery [1-7], and few on the clinical features of ESCC patients with clinically diagnosed IM by endoscopy. Approximately half of the pathological IM cases were identified during preoperative examination [6,7], and the characteristics and outcomes of ESCC patients with clinically diagnosed IM remain unknown.

Multimodal treatment with a significant antitumor effect is required to improve outcomes of patients with IM; however, there is no determined standard treatment for ESCC patients with clinically diagnosed IM. Hokamura et al. evaluated the efficacy of neoadjuvant chemotherapy (NAC) that consisted of cisplatin and 5-fluorouracil (CF) for ESCC with IM diagnosed by endoscopy [8]. They reported no NAC survival benefit when compared with their historical data. However, this study conducted a small-sample single-arm trial, with only 15 patients.

This study aimed to analyze the characteristics of ESCC patients with clinically diagnosed IM and assess their outcomes stratified by the treatment modalities and survival rates to explore the treatment strategy suited for these patients.

## Materials and methods

Patients

A total of 2,772 patients underwent initial endoscopy for ESCC in the Cancer Institute Hospital of Japanese Foundation for Cancer Research (JFCR) between 2005 and 2018. Among them, 85 (3.1%) patients were diagnosed with ESCC with IM by endoscopy. A retrospective review of the patients’ medical records was done and data were obtained, including clinical characteristics, treatment, and patient survival. This study was approved by the Institutional Review Board of JFCR (number 2018-1192) and informed consent was obtained.

Definition of IM

**Figure 1:**
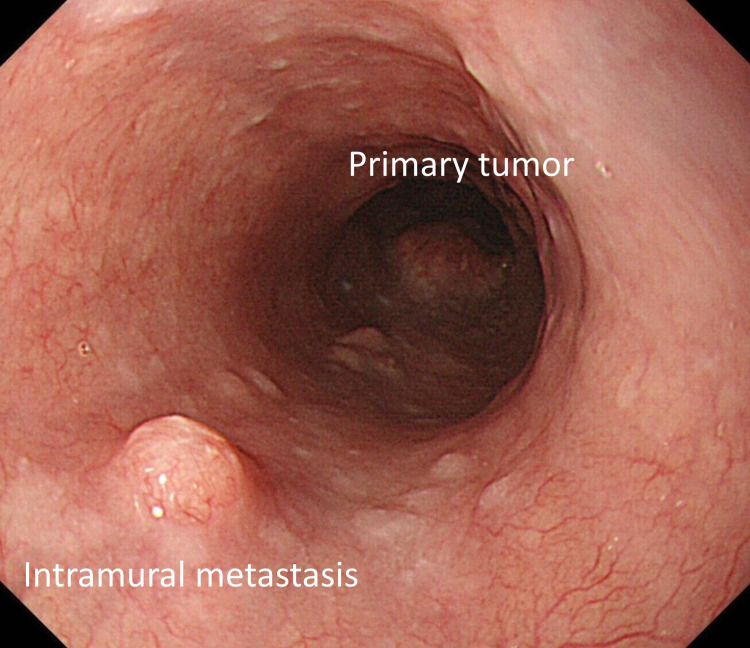
Typical endoscopic finding of intramural metastasis.

Based on the endoscopic findings, clinical IM was characterized by the following (Figure 1): (1) separated from the primary tumor, (2) located in the esophagus or stomach wall, and (3) with the gross appearance of a submucosal tumor without intraepithelial component, occasionally with an erosive change [2]. Accompanied with these findings, the presence of an advanced primary lesion in the esophagus strongly suggests an IM tumor. Biopsies or boring biopsies to confirm cancer cells in the submucosal tumor were not performed routinely. Distinguishing a second primary lesion after it has grown to an elevated and ulcerated tumor is challenging; hence, such tumors from IM were excluded. Pathologically, IM was diagnosed as described in the previous study [7].

Treatment strategy for ESCC

All patients had undergone routine evaluations including endoscopy and computed tomography (CT). In patients showing T2 or higher T stage or possible metastatic nodes on CT, positron emission tomography was done. Endoscopic and endobronchial ultrasounds to evaluate clinical nodal involvement and extent of invasion were not performed routinely. Tumor staging was done, and treatment was decided with the multidisciplinary tumor board. In this study, the tumor stage was determined based on the Union for International Cancer Control TNM Classification of Malignant Tumours, 8th edition [9]. IM within the gastric wall was defined as distant metastasis (M1).

Patients were treated according to the Japan Esophageal Society guidelines as follows [10,11]: upfront surgery (US) for stage I disease, definitive chemoradiotherapy (dCRT) for T4b tumor or refusal to surgery irrespective of the stage, and salvage surgery for dCRT failure. However, during this study, treatment for stage II or III disease has changed owing to the result of the randomized trial JCOG9907 [12], and neoadjuvant chemotherapy (NAC) was introduced in 2009. NAC consists of two courses of CF: 80 mg/m2 cisplatin was given on day one and 800 mg/m2/day 5-fluorouracil on days one to five. Before that, NAC was not available. In the case of the tumor suspected to invade adjacent organs, but not definitely diagnosed as T4b disease, neoadjuvant chemoradiotherapy (NACRT) with CF and 40Gy irradiation was introduced. In this study, US, NAC, NACRT, and dCRT were defined as curative-intent treatment and chemotherapy alone, radiation alone, chemotherapy with palliative radiation, and best supportive care as palliative treatment, respectively. Supraclavicular lymph node metastases (M1) or IM within the gastric wall were not the contraindications of surgery.

Statistical analysis

All data are presented as median (range) or number (%). The Mann-Whitney U test and Fisher’s exact test were appropriately used for statistical comparisons between groups. The overall survival was for the period from the date of diagnosis to either death or the last follow-up, and survival analysis was performed using the Kaplan-Meier method and log-rank test. A Cox proportional hazard model was used to evaluate the impact of variables on survival, and the hazard ratios (HRs) and 95% confidence intervals (CIs) were obtained. In the multivariate analysis, we included potential confounders, including sex, age, performance status, initial clinical T stage, N stage, M stage, and the treatment modalities. All statistical analyses were performed using the SPSS software package (version 23.0; IBM Corp., Armonk, NY). A P-value < 0.05 was considered statistically significant.

## Results

Patient characteristics and location of IM

Table 1 summarizes the patient characteristics. Of 85 patients, 76 (89.4%) had T3 or T4 tumors, 73 (85.9%) had nodal metastases, and 36 (42.4%) had M1 diseases. Among these M1 patients, there were supraclavicular lymph node metastasis in 20 (55.6%), IM within the gastric wall in 11 (30.6%), distant organ metastasis in 10 (27.8%), and distant lymph node metastasis in five (13.9%) patients. Curative-intent treatment could be introduced in 63 patients (74.1%). Among them, US, NAC, NACRT, and dCRT were chosen as the initial treatment for 17 (27.0%), 27 (42.9%), 2 (3.2%), and 17 patients (27.0%), respectively. dCRT was more frequently selected for T4 tumors, compared with US or NAC (P = 0.02, Table 2).

**Table 1:** Patient characteristics and treatment modalities. Values are presented as median (range) or n (%). ASA-PS: American Society of Anesthesiologists-Performance Status; US: upfront surgery; NAC: neoadjuvant chemotherapy; NACRT: neoadjuvant chemoradiotherapy; dCRT: definitive chemoradiotherapy.

Variables		Values
Age (years)		67 (31-84)
Gender	Male	71 (83.5)
	Female	14 (16.5)
ASA-PS	Class 1-2	77 (90.6)
	Class 3	8 (9.4)
Main tumor location	Upper	16 (18.8)
	Middle	50 (58.8)
	Lower	19 (22.4)
cT category	cT 1-2	9 (10.6)
	cT 3	59 (69.4)
	cT 4	17 (20.0)
cN category	cN 0	12 (14.1)
	cN 1-3	73 (85.9)
cM category	cM 0	49 (57.6)
	cM 1	36 (42.4)
Initial treatment		
Curative-intent		63 (74.1)
	US	17 (27.0)
	NAC	27 (42.9)
	NACRT	2 (3.2)
	dCRT	17 (27.0)
Palliative		22 (25.9)

**Table 2:** Patient characteristics and major modalities of curative-intent treatment. Data are presented as median (range) or n (%). * P < 0.05. US: upfront surgery; NAC: neoadjuvant chemotherapy; dCRT: definitive chemoradiotherapy; ASA-PS: American Society of Anesthesiologists-Performance Status.

Variables		US (n = 17)	NAC (n = 27)	dCRT (n = 17)	P-value
Age (years)		70 (31-81)	65 (51-78)	67 (45-75)	0.247
Gender	Male	14 (82.4)	23 (85.2)	14 (82.4)	0.957
	Female	3 (17.6)	4 (14.8)	3 (17.6)	
ASA-PS	Class 1-2	15 (88.2)	24 (88.9)	16 (94.1)	0.810
	Class 3	2 (11.8)	3 (11.1)	1 (5.9)	
Main tumor location	Upper	3 (17.6)	2 (7.4)	6 (35.3)	0.210
	Middle	10 (58.8)	16 (59.3)	8 (47.1)	
	Lower	4 (23.5)	9 (33.3)	3 (17.6)	
cT category	cT 1-3	14 (82.4)	26 (96.3)	11 (64.7)	0.022^*^
	cT 4	3 (17.6)	1 (3.7)	6 (35.3)	
cN category	cN 0	3 (17.6)	4 (14.8)	4 (23.5)	0.911
	cN 1-3	14 (82.4)	23 (85.2)	13 (76.5)	
cM category	cM 0	11 (64.7)	20 (74.1)	10 (58.8)	0.558
	cM 1	6 (35.3)	7 (25.9)	7 (41.2)	

Regarding IM, 39 (45.9%) patients had IM on the oral side of the primary tumor, 40 (47.1%) had IM on the anal side, and six (7.1%) had IM on both sides. IM within the gastric wall was noted in 13 (15.3%) patients. The size and numbers of IM were not recorded.

Patient survival stratified by initial treatment modalities

**Figure 2:**
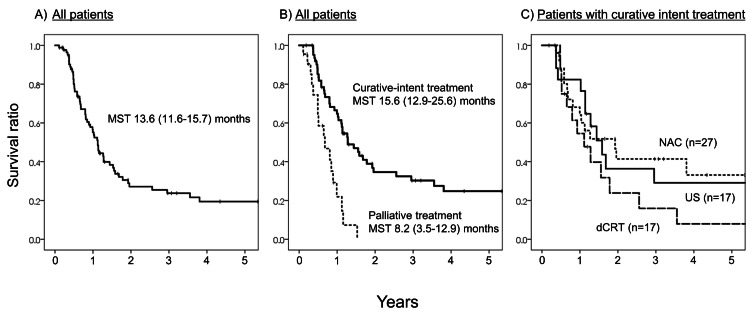
Kaplan-Meier curves. A: Overall survival of 85 patients. B: Overall survival of patients with curative-intent treatment and those with palliative treatment. C: Overall survival among patients with major curative-intent treatment stratified by each treatment modality. MST: median survival time; US: upfront surgery; NAC: neoadjuvant chemotherapy; dCRT: definitive chemoradiotherapy.

Kaplan-Meier curves are shown in Figure 2. A total of 85 patients had a median survival time (MST) of 13.6 months (95% CI: 11.6-15.7). When the curative-intent treatment could not be introduced, MST was 8.2 months (95% CI: 3.5-12.9). When the curative-intent treatment was introduced, MST was 15.6 months (95% CI: 10.7-20.4). The MST of US and NAC patients was 19.3 (95% CI: 12.9-25.6) and 23.4 months (95% CI: 9.4-37.4), respectively, and no significant difference was noted between US and NAC patients (P = 0.89).

Impact of each treatment modality with curative-intent on survival

We investigated the clinical impact of treatment modalities on survival in patients who received curative-intent treatment, excluding NACRT patients (Table 3). In a univariate analysis, HR of NAC was 0.95 (95% CI: 0.43-2.06, P = 0.89) when compared with US. After multivariate adjustment, NAC had no significant benefit for OS when compared with US (HR: 0.98, 95% CI: 0.39-2.45, P = 0.96).

**Table 3:** Clinical impact of variables on overall survival in patients with curative-intent treatment. * P < 0.05. HR: hazard ratio; CI: confidence interval; ASA-PS: American Society of Anesthesiologists-Performance Status; US: upfront surgery; NAC: neoadjuvant chemotherapy; dCRT: definitive chemoradiotherapy.

Variables		Unadjusted HR (95% CI)	P-value	Adjusted HR (95% CI)	P-value
Age	<75	1	-	1	-
	≥75	2.130 (0.74-6.17)	0.163	2.06 (0.59-7.27)	0.260
Gender	Female	1	-	1	-
	Male	1.42 (0.55-0.36)	0.469	1.43 (0.54-3.83)	0.472
ASA-PS	Class 1-2	1	-	1	-
	Class 3	3.12 (1.20-8.10)	0.019*	3.72 (1.23-11.3)	0.020*
cT category	cT 1-3	1	-	1	-
	cT 4	1.63 (0.77-3.43)	0.201	1.38 (0.53-3.59)	0.516
cN category	cN 0	1	-	1	-
	cN 1-3	1.53 (0.60-3.92)	0.373	1.24 (0.45-3.42)	0.682
cM category	cM 0	1	-	1	-
	cM 1	0.81 (0.59-1.12)	0.199	0.83 (0.58-1.18)	0.287
Initial treatment	US	1	-	1	-
	NAC	0.95 (0.43-2.06)	0.888	0.98 (0.39-2.45)	0.959
	dCRT	1.69 (0.75-3.79)	0.204	1.72 (0.68-4.35)	0.250

Accuracy of clinical diagnosis for IM by endoscopy

Among the 17 US patients who were diagnosed as having IM by endoscopy, three patients (17.6%) did not have IM in pathological findings, indicating that the positive predictive value for clinical diagnosis of IM by endoscopy was 82.4%.

Pathological IM and preoperative treatment in patients who underwent surgery

**Figure 3:**
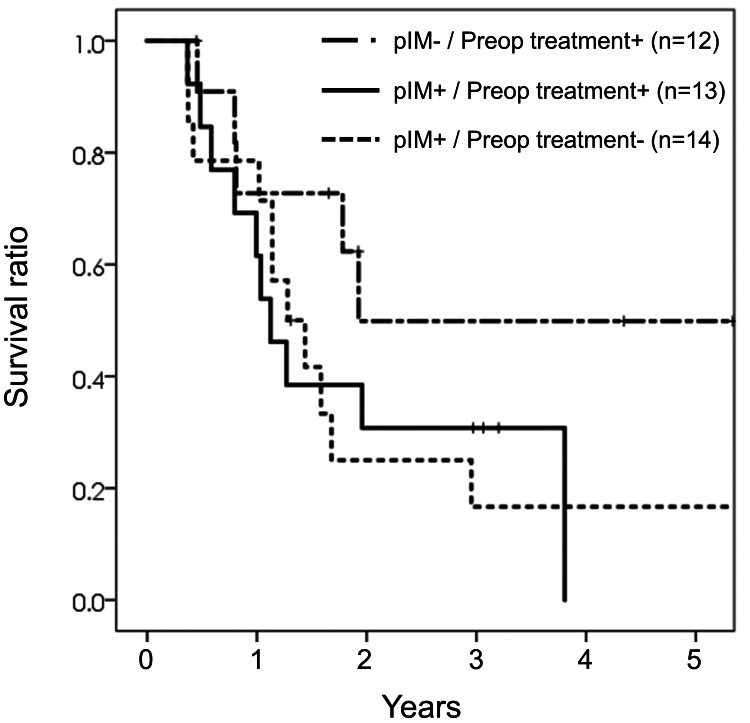
Kaplan-Meier curves stratified by pathological IM and preoperative treatment. pIM: pathological intramural metastasis.

Finally, we investigated the clinical impact of pathological IM on survival stratified by preoperative treatment in 42 patients who underwent surgery; 17 of US patients, 22 of NAC, one of NACRT, and two of dCRT, respectively. Patient survivals were calculated in pathological IM-positive patients (n = 13) and IM-negative patients (n = 12) who received preoperative treatment and US patients with pathological IM (n = 14). Despite the pathological IM-negative patients who received preoperative treatment possibly included patients with false-positive clinical IM, they attained long-term survival of approximately 50% (Figure 3). Also, the percentage of patients who did not have pathological IM in the NAC group (12 of 25, 48.0%) was higher than that in the US group (three of 17, 17.6%) significantly (P = 0.044).

## Discussion

In this study, the clinical characteristics of patients with clinically diagnosed IM from ESCC and the impact of treatment modalities on survival were evaluated. It was observed that advanced tumors were more common in these patients, and the prognosis was poor. NAC with CF for these patients had no significant survival benefit when compared with US. Additionally, when US could be performed as a curative-intent treatment, the prognosis might improve compared to palliative treatment. Patients wherein pathological IM was not detected after preoperative treatment attained long-term survival of approximately 50% although they might include those with false-positive clinical IM.

In 1933, Watson firstly reported IM from esophageal cancer as cancer spreading through the submucosal lymphatic system [13]. It was reported that IM from ESCC is one of the important poor prognosticators and the reliable indicator for lymphatic invasion [1-7]. Although the IM in ESCC is not a factor to determine tumor, node, and metastasis (TNM) classification, it suggests highly aggressive biological behavior with a strong prognostic impact. Thus, multimodal treatment with a strong antitumor effect should be given.

However, there is no determined standard treatment for ESCC with IM. Moreover, few studies have focused on patients with a clinical diagnosis of IM from ESCC. Neoadjuvant treatment has become the standard care for patients with resectable advanced esophageal cancer worldwide. However, regarding the efficacy of NAC with CF for resectable ESCC with clinical IM, no significant survival benefit was noted when compared with US. This result was consistent with the data reported by Hokamura et al., who suggested no survival benefit of NAC with CF when compared with their historical data [8]. Moreover, it has been shown that clinical IM is significantly associated with treatment failure in patients wherein NAC with CF was given [14]. Therefore, NAC with CF might be insufficient for these patients. Recently, the JCOG1109 trial showed the superiority of triplet NAC (docetaxel plus CF) over CF in terms of survival [15]. Also, in the CheckMate 577 trial, adjuvant immune checkpoint inhibitor (ICI) offered a better prognosis in ESCC patients who underwent NACRT followed by curative esophagectomy [16]. Henceforth, these promising treatment strategies should be evaluated.

Although NAC presented no significant survival benefit, 48.0% of patients whose pathological IM could not be detected after preoperative treatment achieved long-term survival. In selecting patients eligible for curative surgery and identifying the long-term survivors, chemoselection (selecting surgical candidates after evaluating the response to treatment) may be efficacious. However, as shown in this study, false clinical IM-positive patients are possibly reported. The percentage of patients who did not have pathological IM in the NAC group (48.0%) was higher than that in the US group (17.6%), indicating that true clinical IM might disappear after preoperative treatment in approximately 30% of clinical IM-positive patients. Still, the clinical diagnosis accuracy of IM is not sufficient, and adding endoscopic ultrasonography targeting suspicious IM lesions may improve the diagnostic accuracy.

This study has several limitations. First, this was a retrospective observational study with a limited number of patients in a single institution. Second, as mentioned above, NAC with CF was introduced after 2009, suggesting that change in protocol influenced the results. However, despite advances in treatment strategy and management for ESCC, NAC, which is a recent treatment, did not show a survival benefit for these patients when compared with US. Third, this study was based on the past clinical diagnosis of IM, and the diagnostic accuracy and detailed findings of IM were insufficient. Therefore, a more extensive multicenter cohort study with a more accurate diagnosis of clinical IM is essential to validate our findings.

## Conclusions

The prognosis of patients with a clinical diagnosis of IM from ESCC is poor even when curative-intent treatment is performed. Furthermore, NAC with CF does not contribute to prolonging survival when compared with US. Treatment strategies with more antitumor effects would be required for these patients.
